# Electrochemical detection of illicit sibutramine adulteration in herbal weight-loss products using an AgNPs-ErGO modified electrode

**DOI:** 10.5599/admet.3433

**Published:** 2026-07-05

**Authors:** Nguyen Dang Thuy Anh, Vo Thi Cam Le, Huynh Van Chung, Ngo Thi Thanh Xuan, Hoang Thi Minh Nguyet, Pham Thi Thanh Ha, Ha Thuy Trang, Le Hoang Hao, Dao Thi Cam Minh, Nguyen Hai Phong, Dinh Quang Khieu

**Affiliations:** 1 University of Medicine and Pharmacy, Hue University, Vietnam; 2 Hanoi University of Pharmacy, Vietnam; 3 Buon Ma Thuot Medical University, Vietnam; 4 Drug, cosmetic and food quality control center of Hue city, Vietnam; 5 Hue Central Hospital, Vietnam; 6 University of Education, Hue University, Vietnam; 7 University of Science, Hue University, Vietnam

**Keywords:** AgNPs-ErGO nanocomposite, electrochemical sensor, sibutramine detection, linear sweep voltammetry, one-step electrochemical synthesis, herbal product adulteration

## Abstract

**Background and purpose:**

Sibutramine is a synthetic anti-obesity drug that has been banned or restricted in many countries due to serious cardiovascular risks. However, its illegal adulteration in herbal weight-loss products remains a public health concern. This study aimed to develop a simple, sensitive, and reliable electrochemical method for the determination of sibutramine in herbal weight-loss products using a silver-nanoparticle-electrochemically reduced graphene oxide modified glassy carbon electrode.

**Experimental approach:**

Graphene oxide, electrochemically reduced graphene oxide, silver nanoparticles and AgNPs-ErGO nanocomposite were synthesized and characterized using X-ray diffraction, Fourier-transform infrared spectroscopy, scanning electron microscopy combined with energy dispersive X-ray spectroscopy, Raman spectroscopy and high-resolution transmission electron microscopy. The AgNPs-ErGO-modified glassy carbon electrode was fabricated and used for sibutramine detection by linear sweep voltammetry. Experimental parameters affecting the analytical signal were optimized. The method was validated according to AOAC guidelines and compared with an HPLC-DAD reference method.

**Key results:**

The AgNPs-ErGO/GCE exhibited improved electrochemical response toward sibutramine compared with bare and other modified electrodes. The developed method showed good selectivity, linearity, precision, accuracy, and sensitivity for sibutramine determination. Application to commercial herbal weight-loss samples demonstrated the suitability of the proposed method for real sample analysis, with results comparable to those obtained by HPLC-DAD.

**Conclusion:**

The AgNPs-ErGO/ /GCE-based electrochemical method provides a rapid, cost-effective, and reliable approach for detecting sibutramine adulteration in herbal weight-loss products. This work contributes to the development of practical analytical tools for quality control and consumer safety monitoring.

## Introduction

In recent decades, obesity has emerged as a global epidemic, with a rapidly increasing prevalence worldwide and strong associations with chronic diseases such as diabetes, cardiovascular disorders, and metabolic syndrome. Consequently, weight management has become a major health concern, leading to the widespread consumption of weight-loss products, including herbal-based supplements and functional foods. These products are often marketed as “natural” and safe; however, numerous studies have reported the adulteration of such formulations with undeclared synthetic pharmaceutical compounds, posing serious risks to public health [1,2]. Among the commonly detected adulterants, sibutramine (SIB), phenolphthalein, caffeine, and fluoxetine are frequently identified [3]. In particular, SIB, a serotonin-norepinephrine reuptake inhibitor (SNRI), was originally developed as an antidepressant and was later approved by the U.S. Food and Drug Administration (FDA) in 1997 for the treatment of obesity [4,5]. However, due to its severe adverse effects, including increased blood pressure, elevated heart rate, and the risk of serotonin syndrome, sibutramine was withdrawn from the market in 2010 [6]. In Vietnam, SIB has been officially banned from health-protective food products under Circular No. 10/2021/TT-BYT [7]. Despite strict regulations, the illegal addition of sibutramine into herbal weight-loss products remains prevalent, primarily driven by the desire to enhance rapid weight-loss efficacy. To address this issue, various analytical techniques have been developed for the detection and quantification of SIB, including reversed-phase high-performance liquid chromatography (RP-HPLC) [8], HPLC [9], high-performance liquid chromatography-tandem mass spectrometry (LC-MS/MS) [10], gas chromatography-MS (GC-MS) [1,11] and electrochemical analysis [12]. While chromategraphic and mass spectrometric methods provide high sensitivity and accuracy, they typically require expensive instrumentation, sophisticated operation, and time-consuming sample preparation, thereby limiting their applicability to rapid, on-site screening. Simpler techniques, such as spectrophotometry or capillary electrophoresis, are lower-cost but often have limited sensitivity and selectivity, particularly in complex matrices such as herbal formulations. In recent years, electrochemical methods have gained considerable attention as promising alternatives for pharmaceutical analysis due to their inherent advantages, including simplicity, low cost, rapid response, high sensitivity, and potential for miniaturization and portable applications. Importantly, the analytical performance of electrochemical sensors can be significantly enhanced by modifying the electrode surface with nanostructured materials. Graphene-based materials, especially reduced graphene oxide (rGO), have been widely employed for their excellent electrical conductivity, large specific surface area, and strong adsorption capacity [13]. However, pristine rGO still suffers from certain limitations, such as restacking of graphene layers and a limited number of active catalytic sites, which restrict its electrocatalytic performance. On the other hand, metal nanoparticles, particularly silver nanoparticles (AgNPs), exhibit outstanding catalytic activity and can effectively accelerate electron-transfer kinetics [14]. Nevertheless, AgNPs alone tend to aggregate and lack structural stability when directly applied as electrode modifiers. To overcome these drawbacks, the integration of AgNPs with rGO into a hybrid nanocomposite provides a synergistic effect, in which rGO serves as a conductive and high-surface-area support to disperse AgNPs, while AgNPs act as active catalytic centres that enhance the electrochemical oxidation of target analytes. Therefore, in this study, a novel electrochemical sensor based on a glassy carbon electrode (GCE) modified with silver nanoparticles-electrochemically reduced graphene oxide (AgNPs-ErGO) was developed for the determination of sibutramine in herbal-based weight-loss products. The proposed sensor exploits the synergistic interaction between AgNPs and ErGO to enhance electrocatalytic activity and analytical performance. The method was systematically optimized and validated, and its applicability was demonstrated by analysing real samples collected from the market. The obtained results were further compared with those from a conventional HPLC-DAD method to confirm the reliability and accuracy of the proposed approach.

## Experimental

### Chemicals

Sibutramine hydrochloride monohydrate (purity 98.4 %, CAS No. 125494-59-9) was used as the analytical standard. Graphite powder (Sigma-Aldrich, USA), sulfuric acid (H₂SO₄, 98 %), phosphoric acid (H₃PO₄, 85 %), hydrochloric acid (HCl, 37 %), hydrogen peroxide (H₂O₂, 30 %), potassium chloride (KCl), and other analytical-grade reagents were obtained from Merck (Germany). Potassium permanganate (KMnO₄) was purchased from Scharlau (Spain). Nafion solution (5 wt.%) was supplied by Aldrich. All solutions were prepared using deionized water.

Britton-Robinson (BR) buffer (0.25 M) was prepared from boric acid, phosphoric acid, and acetic acid, and adjusted to the desired pH (2 to 12) using 1.0 M NaOH. Commercial herbal weight-loss products were collected from pharmacies and e-commerce platforms in Vietnam.

### Apparatus

Electrochemical measurements were performed using a PCPA HH5 electrochemical workstation in a conventional three-electrode system consisting of a modified glassy carbon electrode (GCE) as the working electrode, a platinum wire as the counter electrode, and an Ag/AgCl (3 M KCl) electrode as the reference. Material characterization was carried out using X-ray diffraction (Bruker D8 Advance), Fourier-transform infrared (FTIR) spectroscopy (PerkinElmer), scanning electron microscopy (SEM, Hitachi S-4800) coupled with energy-dispersive X-ray mapping, Raman spectroscopy (Horiba Xplora Plus, 785 nm), and high-resolution transmission electron microscopy (JEOL JEM-2100F). For validation, an HPLC with a diode-array detection (HPLC-DAD) system (Shimadzu LC-20AD with a PDA detector) was used for comparison. The HPLC-DAD method was validated in accordance with the AOAC guidelines [15] and demonstrated satisfactory analytical performance. The chromatographic conditions were as follows: an InertSustain™ C18 column (4.6×250 mm, 5 μm) was used for separation. The mobile phase consisted of acetonitrile (ACN, solvent A) and KH₂PO₄ buffer solution (pH 3.2, solvent B) under the following gradient elution program: 0 to 1 min, 20 % A; 1 to 3 min, 40 % A; 3 to 11 min, 50 % A; 11 to 12 min, 50 % A; 12 to 13 min, 20 % A and 13 to 28 min, 20 % A. The flow rate was maintained at 0.8 mL min^-1^, the injection volume was 20 μL, and the column temperature was set at 40 °C. Detection was carried out at a wavelength of 225 nm.

### The synthesis of electrochemically reduced graphene oxide, AgNPs and AgNPs-ErGO for material characterization

#### Synthesis of graphene oxide and electrochemically reduced graphene oxide

First, graphene oxide (GO) was synthesized from graphite using a modified Hummers method [16]. Subsequently, electrochemically reduced graphene oxide (ErGO) was synthesized from GO via a one-step electrochemical reduction method [17] according to the following procedure:

A 0.1 M phosphate buffer solution (PBS, pH 7) was prepared and GO powder was dispersed into the PBS solution at a concentration of 1 mg mL^-1^. The mixture was ultrasonicated for 2 h to obtain a homogeneous GO-PBS suspension. Stainless steel mesh (Inox 304) was used as the working electrode. Prior to use, the mesh was cleaned by ultrasonication in 2 M HNO₃ and 96 % ethanol to remove impurities and the native oxide layer and then dried. The electrochemical synthesis was carried out in a conventional three-electrode system consisting of the stainless steel mesh as the working electrode, a platinum wire as the counter electrode and an Ag/AgCl|3 M KCl electrode as the reference electrode. The stainless steel mesh was immersed in the GO-PBS suspension. The electrochemical reduction and deposition of ErGO were performed by cyclic voltammetry (CV) in the potential range from 0 to -2.0 V at a scan rate of 0.100 V s^-1^ for 10 consecutive cycles.

After electrochemical deposition, the ErGO-coated stainless steel mesh was removed and immersed in distilled water. The porous ErGO film was completely detached from the mesh surface by ultrasonication for approximately 15 min. To completely remove residual salt ions from the buffer solution, the dispersed ErGO suspension was centrifuged at 6000 rpm, the supernatant was discarded, and the solid was washed three times with distilled water. Finally, the purified ErGO product was freeze-dried to obtain a lightweight porous powder, which was then ground into a fine powder for further characterization.

#### Synthesis of silver nanoparticles

The synthesis of AgNPs was carried out electrochemically using a three-electrode system as follows: Stainless steel mesh (Inox 304) was used as the working electrode and was cleaned by ultrasonication in 2 M HNO₃ and 96 % ethanol, followed by drying. A solution containing 50 mL of 0.1 M KNO₃ as the supporting electrolyte and 0.05 M AgNO₃ as the silver precursor was prepared in the electrochemical cell.

The electrochemical synthesis was performed using a three-electrode configuration comprising the stainless steel mesh working electrode, platinum wire counter electrode, and Ag/AgCl|3 M KCl reference electrode. The reduction and deposition process was carried out by cyclic voltammetry over the potential range from 0 to -0.6 V at a scan rate of 0.100 V s^-1^ for 10 consecutive cycles.

After the reduction process, the Inox 304 mesh electrode was removed from the electrochemical cell and rinsed thoroughly with distilled water to eliminate residual AgNO₃. The electrode was then immersed in distilled water and ultrasonicated for approximately 15 min to detach the deposited AgNPs layer from the mesh surface. During this process, the solution gradually turned yellowish-brown, while the mesh regained its metallic appearance.

The obtained suspension was centrifuged at 6000 rpm, the supernatant was discarded, and the precipitate was washed three times with distilled water. The purified AgNPs were finally freeze-dried and used for further characterization.

#### Synthesis of AgNPs-GO and AgNPs-ErGO

The AgNPs-GO composite was prepared by mechanically mixing AgNPs and GO at a mass ratio of 1:5. The AgNPs-ErGO composite was synthesized electrochemically using a three-electrode system according to the following procedure: Stainless steel mesh (Inox 304) was cleaned as described above and used as the working electrode. A precursor suspension was prepared directly in the electrochemical cell by mixing 1.8 mL of 0.05 M AgNO₃, 48.2 mL of GO suspension (1.0 mg mL^-1^) and 0.51 g of KNO₃ as the supporting electrolyte. These quantities were selected to obtain an AgNPs:GO mass ratio of approximately 1:5, assuming the complete electrochemical reduction of Ag⁺ ions to metallic Ag nanoparticles. The electrochemical synthesis was then carried out using a conventional three-electrode system consisting of a stainless steel mesh working electrode, a platinum wire counter electrode, and an Ag/AgCl reference electrode. The reduction and deposition process was performed by cyclic voltammetry over the potential range from 0 to -2.0 V at a scan rate of 0.100 V s^-1^ for 10 consecutive cycles. During this process, both the oxygen-containing functional groups on GO and Ag⁺ ions were simultaneously reduced, leading to the formation of an AgNPs-ErGO nanocomposite film on the stainless steel mesh surface. The immersed portion of the stainless steel mesh gradually turned dark grey, confirming the successful deposition of the AgNPs-ErGO film.

After electrochemical reduction, the modified mesh electrode was removed and rinsed with distilled water. The electrode was dried at 60 °C for 2 h to remove residual water and promote stronger anchoring of AgNPs within the folded ErGO network. Subsequently, the mesh was immersed in distilled water and ultrasonicated for approximately 15 min to detach the AgNPs-ErGO film from the mesh surface.

The obtained suspension was centrifuged at 6000 rpm, washed three times with distilled water, and finally freeze-dried to obtain purified AgNPs-ErGO powder for material characterization.

### Preparation of modified electrodes for electrochemical measurements

*Bare GCE*: The surface of the glassy carbon electrode (GCE) was polished using 0.05 μm Al₂O₃ powder until a mirror-like surface was obtained. The polished GCE was then immersed in 2 M HNO₃ solution for 15 min. Subsequently, the electrode was rinsed twice with 96 % ethanol and distilled water, with each wash repeated 3 times. Finally, the electrode was dried naturally at room temperature.

*GO/GCE electrode*: GO suspension was prepared by dispersing 0.1 g of GO in 100 mL of distilled water followed by ultrasonication for 2 h to obtain a homogeneous GO suspension (1.0 mg mL^-1^). Then, 400 μL of 1% nafion solution (used as a binder) was added to 10 mL of the GO suspension. Afterward, V μL of the obtained mixture was drop-cast onto the cleaned GCE surface and dried under an infrared lamp until complete solvent evaporation, yielding the GO/GCE electrode.

*ErGO/GCE electrode*: 10 mL of the synthesized ErGO suspension was mixed with 400 μL of 1 % nafion solution. Subsequently, V μL of the mixture was drop-cast onto the cleaned GCE surface and dried under an infrared lamp.

*AgNPs/GCE electrode*: 10 mL of AgNPs suspension (1 mg mL^-1^) was mixed with 400 μL of 1 % nafion solution. Then, V μL of the resulting suspension was drop-cast onto the cleaned GCE surface and dried under an infrared lamp.

*AgNPs-GO/GCE electrode*: AgNPs were mechanically mixed with GO at an appropriate mass ratio. Subsequently, 0.1 g of the obtained composite material was dispersed into 100 mL of distilled water to form an AgNPs-GO suspension (1 mg mL^-1^). Then, 400 μL of 1 % nafion solution was added to 10 mL of the AgNPs-GO suspension. Finally, V μL of the resulting mixture was drop-cast onto the cleaned GCE surface and dried under an infrared lamp.

*AgNPs-ErGO/GCE electrode*: 10 mL of the AgNPs-ErGO composite suspension was mixed with 400 μL of 1% Nafion solution. Subsequently, V μL of the mixture was drop-cast onto the cleaned GCE surface and dried under an infrared lamp to obtain the AgNPs-ErGO/GCE modified electrode.

### Placebo preparation

The placebo sample used in this study was Slimtosen slimming capsules (0.5790 g capsule^-1^), containing herbal ingredients including *Folium Nelumbinis*, *Alisma plantago*, *Gynostemma pentaphyllum*, *Garcinia cochinchinensis*, *Cassia tora L*., chitosan, L-carnitine fumarate, and other excipients. Ten capsules were randomly selected, and their entire powder contents were thoroughly homogenized in an agate mortar for 2 h.

The placebo sample preparation procedure was performed as follows: 0.5 g of the homogenized powder was accurately weighed into a 50 mL Falcon tube. Subsequently, 25 mL of n-hexane acidified with HCl was added, and the mixture was shaken for 5 min to remove nonpolar interfering substances. After discarding the supernatant, the remaining residue was collected and dried at room temperature. Then, 25 mL of absolute methanol was added to the dried residue, followed by vortex mixing for 5 min and ultrasonication for 10 min to facilitate analyte extraction. The resulting clear solution was filtered through a 0.45 μm membrane filter prior to analysis. Three placebo samples were independently prepared following the same procedure.

## Results and discussion

### Electrochemical synthesis of materials

#### Electrochemical preparation of electrodes

**Figure 1.**
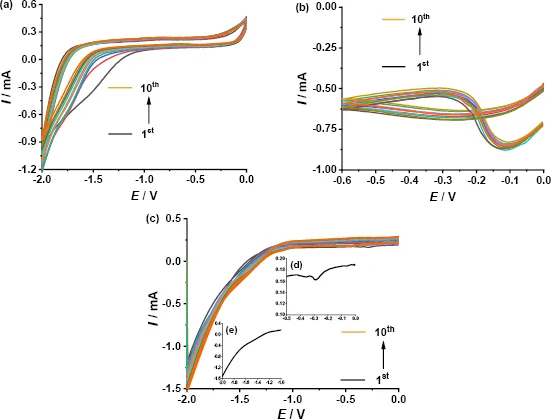
10 consecutive cyclic voltammograms for (a) electrochemical reduction of GO to ErGO; (b) electrodeposition of AgNPs and (c) reduction of Ag⁺ and GO to form AgNPs-ErGO composite . Inset: Magnified views of the cathodic scan showing the reduction of Ag⁺ ions in the potential range of 0.0 to -0.5 V (d) and the reduction of GO in the potential range of -1.0 to -2.0 V (e). Experimental condition for (a) and (c): scan rate: 100 mV s^-1^; step potential: 60 mV

The electrochemical reduction of graphene oxide (GO) to electrochemically reduced graphene oxide (ErGO) was first investigated using cyclic voltammetry (CV). As shown in Figure 1a, a well-defined cathodic peak appears at approximately -1.49 V (*vs.* Ag/AgCl), corresponding to the irreversible reduction of oxygen-containing functional groups such as epoxy, hydroxyl, and carboxyl moieties on the GO sheets. The rapid decrease in peak intensity from the second cycle onward, accompanied by a slight shift toward more negative potentials, confirms the progressive depletion of reducible oxygen functionalities and the formation of a more stable ErGO structure [18]. The disappearance of the reduction peak in subsequent scans indicates the essentially irreversible nature of the GO reduction process. The electrodeposition of silver nanoparticles (AgNPs) was subsequently examined (Figure 1b). A distinct cathodic peak at approximately -0.12 V is attributed to the reduction of Ag⁺ ions to metallic silver according to: Ag^+^ + e^-^ → Ag^0^. The pronounced crossover between forward and reverse scans suggests a typical nucleation and growth mechanism [19]. During the forward scan, the formation of initial Ag nuclei requires overcoming an energy barrier, resulting in relatively low current. In contrast, during the reverse scan, Ag deposition occurs preferentially on pre-formed nuclei, leading to a significantly enhanced current response. For the co-reduction system (Ag⁺-GO), two cathodic peaks are observed at approximately -0.28 and -1.46 V (Figure 1c), corresponding to the reduction of Ag⁺ and GO, respectively.

This clearly indicates the simultaneous formation of AgNPs and ErGO. Compared with the individual systems, the reduction peaks become broader and less well-defined, which can be attributed to competitive adsorption and diffusion processes between GO sheets and Ag⁺ ions. The negatively charged GO sheets facilitate the elec-trostatic adsorption of Ag⁺, promoting nucleation and resulting in a homogeneous distribution of AgNPs on the ErGO matrix. This synergistic electrochemical process enables the *in situ* fabrication of the AgNPs-ErGO composite.

#### The electrochemical preparation of materials

**Figure 2.**
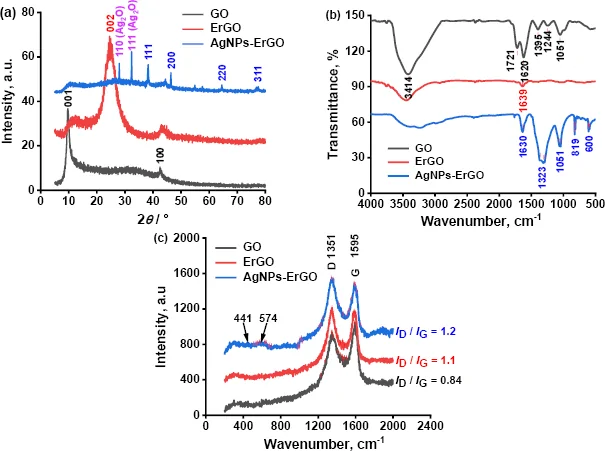
(a) XRD patterns; (b) FT-IR spectra; (c) Raman spectra of GO, ErGO and AgNPs-ErGO

The crystalline structures of GO, ErGO, and AgNPs-ErGO were characterized by X-ray diffraction (Figure 2a). The GO sample exhibits a characteristic diffraction peak at 2*θ* ≈ 9.7°, assigned to the (001) plane, indicating an increased interlayer spacing due to oxygen functionalization. A weak peak at ~42.5° corresponds to the (100) plane of partially retained graphitic domains [20]. After electrochemical reduction, the (001) peak significantly diminishes and shifts to higher angles, while a broad peak emerges at ~24.6°, corresponding to the (002) plane of graphitic carbon [13,21]. This change indicates partial restoration of the sp^2^ carbon network and reduction of interlayer spacing. In the AgNPs-ErGO composite, additional diffraction peaks at 38.3, 44.5, 64.5 and 77.5° are observed, corresponding to the (111), (200), (220) and (311) planes of face-cantered cubic (fcc) silver (JCPDS 04-0783). The coexistence of these sharp peaks with the broad ErGO peak confirms the successful deposition of crystalline AgNPs onto the graphene matrix. Minor peaks associated with Ag₂O suggest slight surface oxidation of AgNPs.

FT-IR spectra (Figure 2b) provide insight into the surface chemistry and reduction process. GO shows characteristic absorption bands at ~3414 cm^-1^ (O-H stretching), 1721 cm^-1^ (C=O stretching), and 1051 to 1244 cm^-1^ (C-O vibrations), confirming a highly oxidized structure [22].

After reduction to ErGO, the intensity of oxygen-related bands significantly decreases, particularly the disappearance of the C=O peak, indicating effective removal of oxygen functionalities and restoration of conjugated carbon domains.

For the AgNPs-ErGO composite, slight shifts and intensity changes in characteristic bands are observed, suggesting interactions between AgNPs and residual oxygen-containing groups. These groups act as anchoring sites, stabilizing AgNPs and preventing aggregation.

Raman spectra (Figure 2c) show two prominent bands: the D band (~1351 cm^-1^) and the G band (~1595 cm^-1^). The *I*_D_/*I*_G_ ratio increases from 0.84 (GO) to 1.10 (ErGO) and further to 1.20 (AgNPs-ErGO), indicating an increase in structural defects and disorder [13, 22].

This increase is associated with the removal of oxygen groups and the creation of new defect sites during reduction. The further increase in the composite suggests that the incorporation of AgNPs introduces additional defect sites or enhances Raman scattering. These defects are beneficial for electrochemical applications, as they provide active sites for electron transfer.

**Figure 3.**
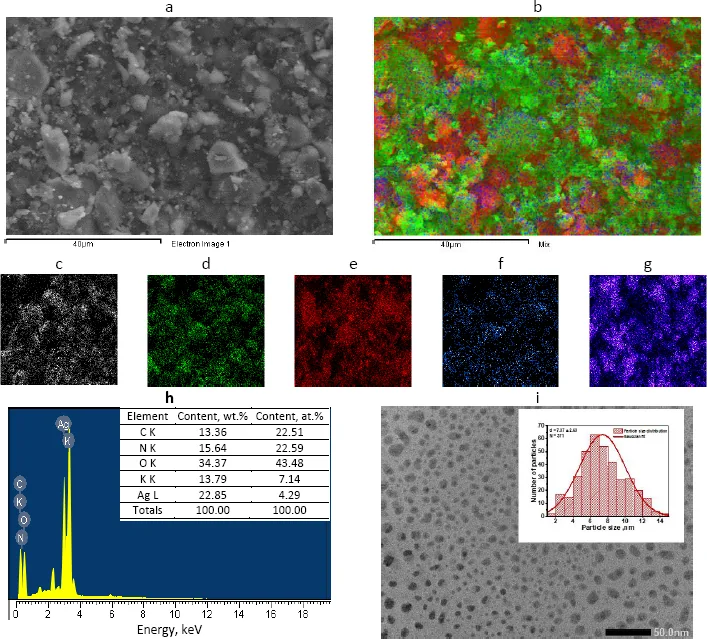
Morphological and elemental analysis of AgNPs-ErGO: a - SEM image; b - mapping image; elemental mapping images of: c - C, d - O, e - Ag, f - N and g - K; h - EDX spectrum and i - TEM image and particle size distribution histogram

The morphology and elemental composition of the synthesized AgNPs-ErGO composite were investigated by EDX-mapping and TEM analyses (Figure 3). The elemental mapping images (Figures 3c to 3g) demonstrate the uniform distribution of C, O and Ag throughout the composite structure, confirming the homogeneous formation of the AgNPs-ErGO material.

The strong carbon signal originates from the graphene framework, while the oxygen signal suggests that a small number of oxygen-containing functional groups remain after the electrochemical reduction process. The silver signal is clearly and uniformly dispersed on the graphene surface, demonstrating that Ag nanoparticles were successfully immobilized without severe agglomeration. The EDX spectrum (Figure 3h) further confirms the elemental composition of the composite. The presence of intense Ag peaks with a relatively high content (22.85 wt.%) indicates the successful incorporation of silver nanoparticles into the ErGO matrix. The strong C signal confirms the graphene-based structure, whereas the O signal is associated with residual oxygen functionalities remaining on partially reduced graphene sheets. In addition, the presence of N and K may originate from residual impurities or precursor species that remain after synthesis and electrochemical treatment. The TEM image (Figure 3i) provides more detailed structural information about the AgNPs-ErGO composite. Transparent and thin graphene sheets with folded and corrugated structures are clearly observed, while dark spherical nanoparticles corresponding to AgNPs are distributed on the ErGO surface. The nanoparticles exhibit relatively uniform dispersion with nanoscale dimensions, suggesting that the ErGO sheets effectively prevent AgNPs aggregation and provide abundant nucleation sites during the deposition process [23]. Such a structural configuration is beneficial for electrochemical applications because the conductive ErGO network facilitates rapid electron transfer, whereas AgNPs provide abundant active catalytic sites, thereby enhancing the electrochemical sensing performance of the modified electrode.

### Investigation and validation of the voltammetric method using a modified electrode for the determination of sibutramine adulterated in herbal weight-loss products

#### Investigation and selection of the modified electrode

**Figure 4.**
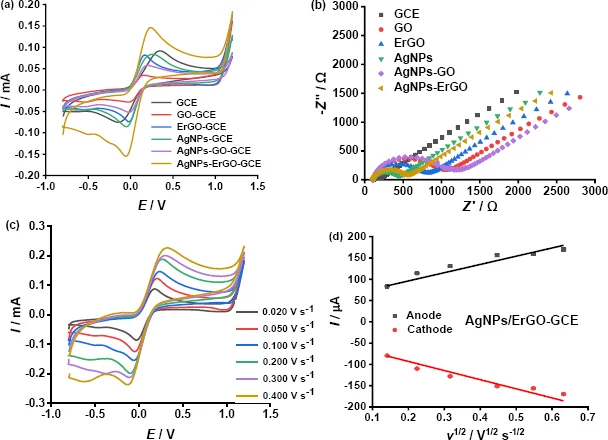
(a) Cyclic voltammograms and (b) Nyquist plots of six different electrodes: bare GCE, GO/GCE, ErGO/GCE, AgNPs/GCE, AgNPs-GO/GCE, and AgNPs-ErGO/GCE recorded in 5 mM [Fe(CN)₆]^3-^/^4-^ containing 0.1 M KCl. (c) cyclic voltammograms of AgNPs-ErGO/GCE at different scan rates and (d) corresponding linear plots of anodic and cathodic peak currents *vs. v*^1/2^. Experimental conditions: potential range from -0.8 to +1.2 V and scan rate of 0.1 V s^-1^

Six types of electrodes were investigated in this study, including bare glassy carbon electrode (GCE), graphene oxide-modified GCE (GO/GCE), electrochemically reduced graphene oxide-modified GCE (ErGO/GCE), AgNPs-modified GCE (AgNPs/GCE), AgNPs-GO-modified GCE (AgNPs-GO/GCE) and AgNPs-ErGO-modified GCE (AgNPs-ErGO/GCE). The selection of a suitable working electrode was evaluated based on electron-transfer capability and electrochemically active surface area using the reversible redox probe [Fe(CN)₆]^3-^/^4-^ (5 mM) in 0.1 M KCl solution (Figures S1 and S2, Supplementary material). The CV and EIS results of the six electrodes are presented in Figures 4a and 4b.

As observed, the oxidation-reduction peak currents varied significantly with the modification layer on the GCE surface. Among them, GO/GCE exhibited the lowest peak current, whereas AgNPs-ErGO/GCE showed the highest current response. Electrodes containing ErGO, including ErGO/GCE and AgNPs-ErGO/GCE, displayed significantly higher peak currents than those containing unreduced GO. This result indicates that the reduction of GO to ErGO substantially improved the electrical conductivity of the material. To quantitatively evaluate the electrochemical performance of the electrodes, CV measurements were performed at scan rates of 0.020 to 0.400 V s^-1^ (Figure 4c). The linear relationships between the anodic/cathodic peak currents and the square root of scan rate (Figure 4d) were employed to estimate the electrochemically active surface area according to the Randles-Sevcik equation. The calculated values are summarized in Table 1.

**Table 1.** Electrochemically active surface area, *I*_p,a_/*I*_p,c_ ratio, and impedance parameters of the six electrodes

Electrodes	*A* / cm^2^		*I*_p,a_/*I*_p,c_ ± SD / μA	Δ*E*_P_ / V	*R*_ct_ / Ω
	Anode	Cathode			
GCE	0.042	0.043	1.06 ± 0.05	0.40 ± 0.11	128
GO/GCE	0.007	0.007	1.01 ± 0.04	0.16 ± 0.02	953
ErGO/GCE	0.034	0.034	1.02 ± 0.02	0.19 ± 0.05	730
AgNPs/GCE	0.037	0.039	0.93 ± 0.01	0.32 ± 0.11	345
AgNPs-GO/GCE	0.027	0.027	0.95 ± 0.02	0.20 ± 0.04	968
AgNPs-ErGO/GCE	0.045	0.046	1.03 ± 0.02	0.30 ± 0.08	441

It appears partially contradictory in Table 1. For example, the bare GCE exhibited the lowest charge-transfer resistance (*R*_ct_ = 128 Ω), which would normally suggest the fastest electron-transfer process. However, this electrode exhibited the largest peak-to-peak separation (Δ*E*_p_ = 0.40 V), indicating relatively slow electrochemical kinetics during cyclic voltammetry.

In contrast, GO/GCE and ErGO/GCE displayed much larger *R*_ct_ values but significantly smaller Δ*E*_p_ values. This apparent inconsistency arises because EIS, Δ*E*_p_, *I*_p,a_/*I*_p,c_, and electrochemically active surface area (ECSA) reflect different aspects of the electrochemical process and are influenced by different physicochemical factors. The low *R*_ct_ value of bare GCE mainly reflects the excellent intrinsic conductivity and compact surface structure of the glassy carbon electrode under near-equilibrium EIS conditions. However, the relatively smooth and chemically inert surface of bare GCE offers a limited number of electroactive sites, leading to a larger Δ*E*_p_ despite its low interfacial resistance. This interpretation is supported by the *I*_p,a_/*I*_p,c_ ratio of 1.06 ± 0.05, which remains close to unity and indicates acceptable reversibility, although the electron-transfer kinetics during dynamic potential scanning are still relatively slow. For GO/GCE, the situation is opposite. The ECSA dramatically decreased to ~0.007 cm^2^ and the *R*_ct_ increased sharply to 953 Ω due to the intrinsically poor conductivity of GO caused by abundant oxygen-containing functional groups disrupting the conjugated carbon network. Nevertheless, GO/GCE exhibited a remarkably small Δ*E*_p_ value (0.16 V) and an *I*_p,a_/*I*_p,c_ ratio close to unity (1.01 ± 0.04). This suggests that although bulk electron transport through the GO layer is hindered, the oxygenated functional groups and structural defects on GO can promote surface-mediated redox interactions and facilitate apparent electrochemical reversibility during CV measurements. After electrochemical reduction, ErGO/GCE showed partially restored conductivity, reflected by a lower *R*_ct_ value (730 Ω) compared with GO/GCE and an increased ECSA (0.034 cm^2^). Simultaneously, the Δ*E*_p_ remained relatively small (0.19 V) and the *I*_p,a_/*I*_p,c_ ratio stayed close to unity (1.02 ± 0.02), indicating improved reversibility and electron-transfer kinetics. This behaviour can be attributed to the recovery of the π-conjugated sp^2^ carbon network after reduction, which enhances conductivity while preserving defect sites and surface roughness favourable for electrochemical reactions [24]. The incorporation of Ag nanoparticles further complicated the electrochemical behaviour. AgNPs/GCE exhibited a relatively low *R*_ct_ (345 Ω) and a high ECSA, demonstrating the conductive and electrocatalytic role of AgNPs. However, the *I*_p,a_/*I*_p,c_ ratio slightly decreased to 0.93±0.01, suggesting some degree of surface heterogeneity or adsorption effects induced by AgNPs. In AgNPs-GO/GCE, the Δ*E*_p_ remained small (0.20 V) despite the very high *R*_ct_ value (968 Ω), again confirming that Δ*E*_p_ and *R*_ct_ are not directly proportional. Here, the redox-active defect sites and surface functionalities of GO likely improved apparent reversibility, whereas the insulating character of GO still dominated the interfacial resistance measured by EIS [25]. Among all electrodes, AgNPs-ErGO/GCE exhibited the best balance of electrochemical properties, with the highest ECSA (0.045-0.046 cm^2^), an *I*_p,a_/*I*_p,c_ ratio close to unity (1.03 ± 0.02), and moderate Δ*E*_p_ and *R*_ct_ values. The synergistic combination of conductive ErGO sheets and electrocatalytically active AgNPs created a highly accessible electroactive surface that facilitated charge transfer and improved reversibility. Therefore, the apparent contradictions in Table 1 arise because EIS and CV characterize different electrochemical phenomena. *R*_ct_ obtained from EIS mainly reflects interfacial electron-transfer resistance under near-equilibrium conditions, whereas Δ*E*_p_ and *I*_p,a_/*I*_p,c_ are additionally influenced by active surface area, adsorption behaviour, surface heterogeneity, and mass transport during dynamic potential scanning. Consequently, the electrochemical performance of the electrodes should be interpreted based on the combined effects of conductivity, electroactive surface area, and electrocatalytic activity rather than a single parameter alone.

#### Investigation of material ratio and modifier loading

**Figure 5.**
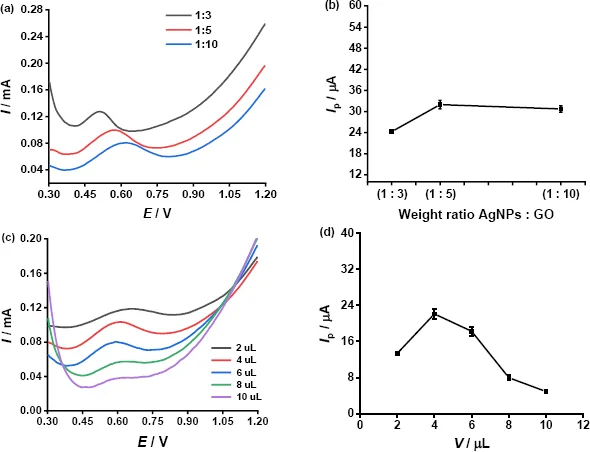
(a) Linear sweep voltammograms of sibutramine obtained at AgNPs-GO modified electrodes with different AgNPs:GO ratios and (b) corresponding variation of oxidation peak current (*I*_P_). (c) LSV responses and (d) variation of oxidation peak current of sibutramine at different loading volumes of AgNPs-GO composite (AgNPs:GO = 1:5, w/w). Experimental conditions: *E*_acc_ (accumulation potential) = -0.2 V, *t*_acc_ (accumulation time) = 120 s, scan rate = 0.100 V s^-1^, 0.05 M BR buffer solution (pH 7) and *C*_SIB_ = 9.9 mg L^-1^

The ratio between AgNPs and GO strongly influenced the electrochemical response toward SIB oxidation (Figures 5a and 5b). As the AgNPs:GO ratio varied from 1:3 to 1:10, noticeable changes in the LSV responses were observed. At the 1:3 ratio, the background current increased significantly because the higher AgNPs content improved the electrical conductivity of the modified layer.

However, the oxidation peak current in SIB did not reach its maximum. Increasing the GO content to a 1:5 ratio resulted in a substantial enhancement in the oxidation peak current, reaching a maximum of approximately 32 μA. This behaviour suggests that the 1:5 ratio provides an optimal balance between the excellent conductivity of AgNPs and the adsorption capability of GO. When the GO content was further increased to 1:10, the peak current slightly decreased, likely due to the restacking of GO sheets, which hindered electron transfer and mass diffusion. Therefore, the AgNPs:GO ratio of 1:5 was selected for further experiments. The effect of modifier loading was also investigated in the range of 2-10 μL (Figure 5c-d). Increasing the deposited volume from 2 to 4 μL significantly increased the oxidation peak current due to the increased number of electroactive sites on the electrode surface. However, when the loading exceeded 4 μL, the current response gradually decreased. This phenomenon can be attributed to the formation of an excessively thick modifier film, which increases diffusion resistance and hinders electron-transfer processes. Consequently, a loading volume of 4 μL was selected as the optimal condition for electrode fabrication.

### Effect of pH and scan rate

**Figure 6.**
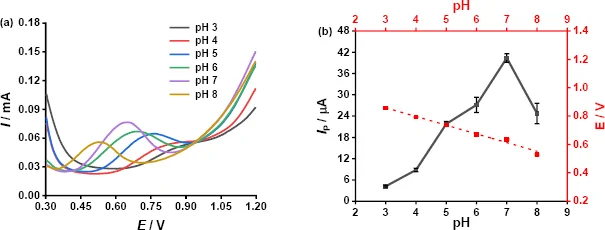
(a) Linear sweep voltammograms of SIB recorded at different pH values, (b) variation of oxidation peak current (*I*_P_) as a function of pH, and (c) linear relationship between oxidation peak potential (*E*_P_) and pH at the AgNPs-ErGO/GCE electrode. Experimental conditions: *E*_acc_ = **-**0.2 V, *t*_acc_ = 120 s, scan rate = 0.100 V s^-1^, 0.05 M BR buffer solution and *C*_SIB_ = 9.9 mg L^-1^

The pH of the supporting electrolyte plays a crucial role in determining the protonation state of SIB and the electron-transfer kinetics at the modified electrode surface (Figure 6a). When the pH increased from 3.0 to 7.0, the oxidation peak current gradually increased, reaching a maximum at pH 7.0 with an Ip of 40.40 ± 0.12 μA. This result indicates that neutral conditions favour the electrochemical oxidation of SIB. Further increasing the pH to 8.0 led to a sharp decrease in current response, which may be associated with changes in the protonation state of SIB and weaker interactions with the electrode surface. Therefore, pH 7.0 was selected as the optimal condition for quantitative analysis (Figure 6b).

In addition, the oxidation peak potential (*E*_p_) shifted linearly toward less positive values with increasing pH, indicating the direct involvement of protons in the electrochemical oxidation process (Figure 6b). The linear regression Equation (1) was obtained.



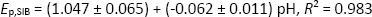

(1)


The slope value (~0.062 V pH^-1^) is close to the theoretical Nernstian value, suggesting that the number of protons and electrons involved in the reaction is equal (*p*/*n* = 1). Therefore, the electrochemical oxidation of SIB at the AgNPs-ErGO/GCE electrode can be considered a proton-coupled electron-transfer process.

**Figure 7.**
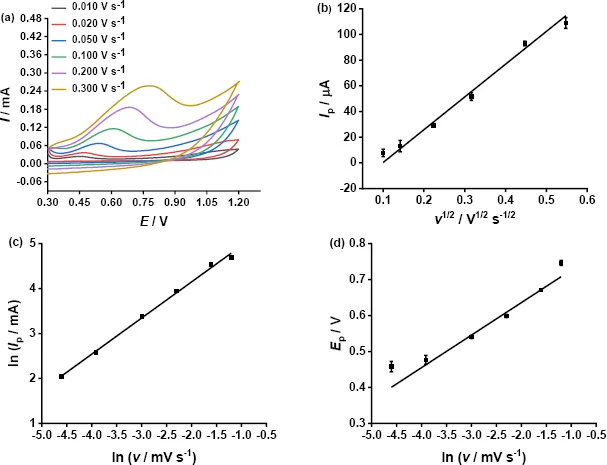
(a) Cyclic voltammograms of SIB at the AgNPs-ErGO/GCE electrode recorded at different scan rates, (b) linear relationship between *I*_P_ and *v*^1/2^, (c) linear plot of ln *I*_P_ vs. ln(*v*) and (d) relationship between *E*_p_ and ln(*v*). Experimental conditions: *E*_acc_ = **-**0.2 V, *t*_acc_ = 120 s, scan rate = 0.100 V s^-1^, 0.05 M BR buffer solution (pH 7) and *C*_SIB_ = 19.6 mg L^-1^

The influence of scan rate on the electrochemical behaviour of SIB was investigated by cyclic voltammetry over the range of 0.010 to 0.300 V s^-1^ (Figure 7a). As the scan rate increased, the oxidation peak current increased, indicating accelerated electrochemical kinetics. A good linear relationship between *I*_p_ and *v*^1/2^ was obtained with *R*^2^ = 0.9901 (Equation 2) (Figure 7b), demonstrating that the mass-transfer process of SIB is strongly influenced by diffusion [26, 27].



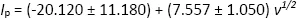

(2)


Moreover, the linear relationship between ln(*I*_p_) and ln(*v*) yielded a slope value of 0.803 (Equation 3) (Figure 7c), which lies between the theoretical values of 0.5 (diffusion-controlled process) and 1.0 (adsorption-controlled process) [28]. This result suggests that the electrochemical oxidation of SIB at the AgNPs-ErGO/GCE electrode follows a mixed diffusion-adsorption controlled mechanism.



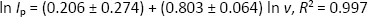

(3)


When investigating the effect of scan rate by cyclic voltammetry (CV) (Figure 7d), the results showed that the electrochemical oxidation of SIB at the modified electrode surface was not governed exclusively by diffusion or adsorption but rather followed a mixed-controlled mechanism involving both processes. However, the adsorption component appeared to play a dominant role. These findings indicate that SIB molecules are not only transported from the bulk solution to the electrode surface by diffusion but also interact with and accumulate on the modified electrode surface prior to electron transfer.

These observations are consistent with trends reported in previous studies. Specifically, Teradal *et al.* [29], in their investigation of SIB oxidation at an ErGO/GCE electrode, observed two irreversible oxidation peaks: the first was adsorption-controlled, whereas the second was diffusion-controlled. Similarly, Lima *et al.* [30], using a screen-printed electrode, reported that the first oxidation peak current of SIB increased linearly with scan rate, indicating that the process was predominantly adsorption-controlled. The authors further emphasized that this mechanism differed from that observed at boron-doped diamond (BDD) electrodes, where the mass-transfer process was mainly diffusion-controlled [31]. In addition, Shi *et al.* [12], using an MIP-rGO modified GCE electrode, reported that the slope of the regression equation between ln *I*_p_ and ln *v* was approximately 0.76, suggesting that the electrochemical oxidation of SIB was controlled by a mixed adsorption-diffusion mechanism.

The large surface area and abundant active sites of ErGO likely facilitate the accumulation of SIB molecules at the electrode surface during the preconcentration step. The electron-transfer coefficient (*α*) was estimated from the relationship between *E*_P_ and *E*_P/2_ (Equation (4)) and was calculated to be approximately 0.47, which is close to the theoretical value of 0.5 for an irreversible electrochemical process [32].



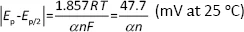

(4)


This result confirms that the oxidation of SIB on the AgNPs-ErGO/GCE electrode is electrochemically irreversible. To determine the heterogeneous electron-transfer rate constant (*k*⁰), it is necessary to calculate the formal redox potential (*E*⁰′). The determination of *E*⁰′ was based on the dependence of the oxidation peak potential (*E*_p_) on the scan rate (*v*). By extrapolating the *E*_p_ *versus v* relationship to *v* = 0, the value of *E*⁰′ can be obtained. Experimental results revealed a nonlinear relationship, Equation (5).



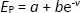

(5)


From this relationship, the formal potential *E*⁰′ was determined to be +0.463 V *vs.* Ag/AgCl|3 M KCl reference electrode.

Subsequently, based on the relationship between *I*_p_ and (*E*_p_ - *E*⁰′), Equation (6) was applied:



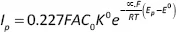

(6)


where *F*: the Faraday constant (96,485 C mol^−1^); *K*^0^: heterogeneous electron-transfer rate constant. Using the values of electrode area *A* = 0.046 cm^2^ and analyte concentration *C*₀ = 58.7 μM, the heterogeneous electron-transfer rate constant *k*⁰ was calculated to be 195.2 s^-1^. This result indicates that the electron-transfer process in SIB at the AgNPs-ErGO/GCE-modified electrode is relatively fast.

Based on the above findings, together with previously reported studies on the electrochemical oxidation of tertiary amine groups [33], the oxidation mechanism of SIB at the AgNPs-ErGO/GCE electrode is proposed as follows. In a BR buffer solution at pH 7, the nitrogen atom of the dimethylamine group uses its lone pair of electrons to bind a proton (H⁺) in solution. Consequently, more than 99 % of SIB molecules exist in the protonated cationic form (SIB-H⁺). This protonated species represents the initial state of the analyte as it approaches the electrode surface.

The investigation of the pH effect on the oxidation peak potential (*E*_P_) mentioned above indicates that the number of electrons and protons involved in the electrochemical reaction is equal. For SIB, this process is typically associated with the oxidation of the tertiary amine group involving the transfer of one electron and one proton.

**Scheme 1.**
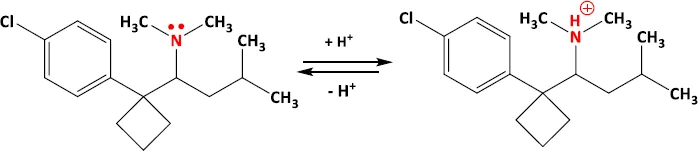
Chemical equilibrium between SIB and protonated SIB-H⁺ in the supporting electrolyte solution

**Scheme 2.**
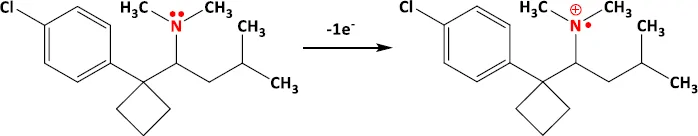
Proposed oxidation process of SIB at the electrode surface

**Scheme 3.**
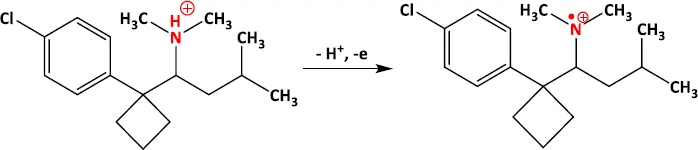
Overall proposed electrochemical oxidation mechanism of SIB at the AgNPs-ErGO/GCE.

To simultaneously satisfy both the Nernstian behaviour (1e⁻/1H⁺) and the initial protonated state of SIB, the oxidation mechanism is proposed to proceed as follows: the reaction initially involves a fast chemical pre-equilibrium step in which the protonated species SIB-H⁺ rapidly dissociates to generate a small amount of neutral free-base SIB along with the release of a proton (H⁺) into the solution (Scheme 1). Subsequently, during the electrochemical step, the nitrogen atom of the free-base SIB donates one electron from its nonbonding lone pair to the electrode surface, leading to the formation of a relatively stable aminium radical cation (N^•^⁺) (Scheme 2). The continuous electrochemical consumption of free-base SIB at the electrode surface drives the chemical equilibrium strongly toward the forward direction. The obtained results, indicating that the electrochemical oxidation of SIB involves the transfer of one electron (1e⁻) and one proton (1H⁺) from the protonated tertiary amine group, are in good agreement with most previously reported studies (Scheme 3), including those published by Freitas *et al.* [31], Sirivibulkovit *et al.* [34], and Kongsuwan *et al.* [35], who proposed similar oxidation mechanisms for SIB using various modified electrodes.

### Optimizing operational parameters

The instrumental parameters, including accumulation potential (*E*_acc_), accumulation time (*t*_acc_), and linear sweep scan rate (*v*_LSV_), were systematically investigated to determine the optimal experimental conditions (Figs. S3-S5). The results revealed that *E*_acc_ = 0 V, *t*_acc_ = 150 s, and *v*_LSV_ = 0.1 V s^-1^ provided the most stable and highest current response. Therefore, these conditions were selected for all subsequent electrochemical measurements.

### Method validation

#### Specificity

**Figure 8.**
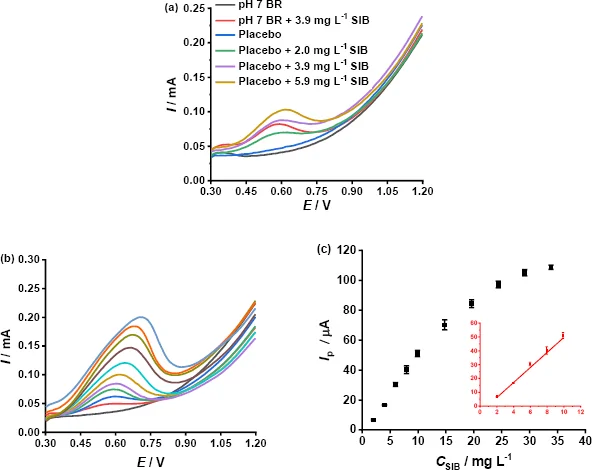
(a) LSVs of the supporting electrolyte, standard SIB solution, placebo sample and placebo samples spiked with different concentrations of SIB under the following experimental conditions: *E*_acc_ = 0 V, *t*_acc_ = 150 s, *v* = 0.100 V s^-1^, 0.05 M BR buffer solution, and pH 7; (b) LSV responses obtained at different SIB concentrations and (c) corresponding calibration plot between *I*_p,SIB_ and *C*_SIB_ (inset: *C*_SIB_ = 2.0 to 9.9 mg L^-1^), experimental conditions: *E*_acc_ = 0 V, *t*_acc_ = 150 s, scan rate = 0.100 V s^-1^, 0.05 M BR buffer solution, pH 7

The voltammetric responses of the supporting electrolyte solution (0.05 M BR buffer, pH 7), placebo sample, and placebo samples spiked with SIB at three concentration levels of 2.0, 3.9 and 5.9 mg L^-1^ were recorded. The obtained results are presented in Figure 8a. In the voltammograms of the supporting electrolyte solution (black curve) and the placebo sample (blue curve), no oxidation peak was observed in the potential range of 0.4-0.8 V. However, after spiking SIB into the placebo matrix at increasing concentrations (from 2.0to 5.9 mg L^-1^), a well-defined oxidation peak clearly appeared at approximately +0.62 V. The oxidation peak current (*I*_p_) increased proportionally with increasing SIB concentration. In addition, the peak obtained was sharp, symmetrical, and completely separated from the background current, indicating good selectivity of the proposed method for SIB detection.

Regarding signal deviation, at C_SIB_ = 2.0 mg L^-1^, the obtained residual standard deviation (RSD) value was 4.93 %, which is lower than the 1/2RSD_H_ value (7.16 %), where RSD_H_ represents the RSD estimated from the Horwitz function. At *C*_SIB_ = 3.9 mg L^-1^, the RSD value was 4.11 %, lower than the corresponding 1/2RSD_H_ value of 6.48 %. Similarly, at C*_S_*_IB_ = 5.9 mg L^-1^, the RSD value was 3.90 %, which was also lower than the corresponding 1/2RSD_H_ value of 6.09 %. At all investigated concentration levels, the experimental RSD values were lower than the corresponding 1/2RSD_H_. These results demonstrate that the proposed method exhibits high selectivity and satisfactory analytical performance for detecting SIB in complex sample matrices [36].

#### Linear range and limit of detection

The concentration of the standard SIB solution in the electrochemical cell was varied from 2.0 mg L^-1^ to 33.8 mg L^-1^. The corresponding linear range investigation is presented in Figure 8b-c. The obtained results demonstrate that the oxidation peak current exhibited a good linear correlation with the concentration of SIB in the range from 2.0 to 14.8 mg L^-1^ according to Equation (7):



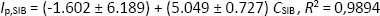

(7)


When the SIB concentration was further increased to 33.8 mg L^-1^, the increase in *I*_P,SIB_ became slower and less significant (Figure 8c), suggesting a tendency toward signal saturation at higher concentrations. However, to accurately establish the linear range of the method, the limit of detection (LOD) and limit of quantification (LOQ) were further evaluated. Since the determination of LOD is typically performed using concentrations close to the origin, the concentration range from 2.0 to 9.9 mg L^-1^ was selected for subsequent analysis (inset of Figure 8c). The corresponding calibration is given by equation (8)



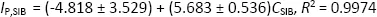

(8)


The LOD and LOQ values were calculated based on the slope of the calibration curve and the standard deviation of the analytical signal. The obtained values were 0.56 mg L^-1^ (or 1.7 μM) for LOD and 1.85 mg L^-1^ for LOQ. The calculated LOD satisfied the conditions of 10 LOD >*C*_min_ and LOQ < *C*_min_ [37]. Therefore, the linear range for the electrochemical determination of SIB was established from 1.85 to 14.8 mg L^-1^.

Table 2 compares the analytical performance of the proposed AgNPs-ErGO/GCE electrode with previously reported electrochemical methods for sibutramine determination. The LOD obtained in the present study (1.7 μM) is higher than those reported for several advanced electrochemical sensors, such as MIP/RGO-GCE (0.02 μM), Na/P-CNTs/GCE (0.03 μM), ERGO/GCE (0.048 μM), and N-GNF@f-CNT/SPE (0.01 μM). The high sensitivity of these previously reported electrodes can be attributed to the use of highly porous nanostructured materials, molecularly imprinted polymers (MIPs), heteroatom-doped carbon materials, or adsorptive stripping techniques, which significantly enhance analyte preconcentration and electron-transfer efficiency.

**Table 2.** Comparison of the LOD and concentration linear range of the proposed electrode with previously reported studies

Electrodes	Method, pH	Linear range, μM	LOD, μM	Sample	Ref.
HMDE	DPV, 4.0	4.2 to 99.6	1.2	Energy drinks, green tea and pharmaceutical formulations	[38]
ERGO/GCE	DPV, 8.0	0.250 to 20.0	0.048	Human serum and urine samples	[29]
BDD	SWV, 0.1 M H₂SO₄	15 to 149.6	0.2 to 5.8	Herbal formulations and dietary supplements samples	[31]
SPE-Gr	AdSDPV, 7,0	2.0 to 120	0.3	Slimming tea beverage	[30]
PGr-ink/GCE	SWAdSV, 8.0	0.04 to 29.9 and 29.9 to 149.6	0.01	Illegal slimming product	[39]
ePAD	SWV, 4.0	7.5 to 250.4	7.4	Capsules, slimming coffee powders and nutraceutical beverages	[34]
Na/P-CNTs/GCE	AdASV, 8.0	0.07 to 59.8 and 59.8 to 179.5	0.03	Weight-loss products	[35]
MIP/RGO-GCE	DPV, 6.5	0.05 to 20	0.02	Human urine and serum samples	[12]
N-GNF@f-CNT/SPE	SWASV, 7.0	0.03 to 29.9 and 29.9 to 179.5	0.01	Food supplements	[40]
AgNPs-ErGO/GCE	LSV, 7.0	5.5 to 44.3	1.7	Herbal weight-loss products	This study

Nevertheless, the proposed AgNPs-ErGO/GCE electrode still delivers satisfactory analytical performance for practical screening applications, particularly for herbal slimming products, where sibutramine is often present at relatively high concentrations. The obtained linear range (5.5 to 44.3 μM) is comparable to or broader than several previously reported methods, including HMDE, BDD and ePAD-based sensors. In addition, although some studies reported lower detection limits, many of those methods require more complicated fabrication procedures, expensive electrode materials, or sophisticated sensing architectures such as molecular imprinting and dual-doped nanocarbon systems.

In contrast, the AgNPs-ErGO/GCE electrode developed in this study offers several practical advantages, including simple preparation, low-cost materials, rapid electrode fabrication through electrochemical synthesis, and satisfactory repeatability and accuracy. The synergistic combination between AgNPs and ErGO effectively improves the electroactive surface area and electron-transfer kinetics, enabling reliable determination of sibutramine in complex herbal matrices. Although it does not offer the lowest LOD among reported methods, the proposed sensor demonstrates strong potential as a simple, rapid, and cost-effective electrochemical platform for routine screening of SIB adulteration in herbal weight-loss products.

#### Intra-day precision

**Figure 9.**
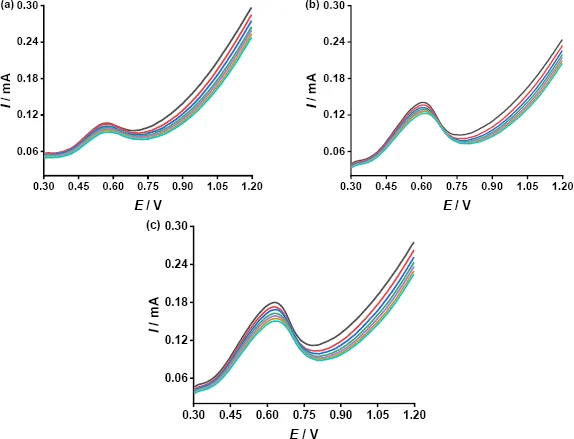
Linear sweep voltammograms of SIB obtained from nine replicate measurements at different concentrations: (a) *C*_SIB_ = 4.0 mg L^-1^, (b) *C*_SIB_ = 9.9 mg L^-1^, and (c) *C*_SIB_ = 15.8 mg L^-1^. Experimental conditions: *E*_acc_ = 0 V, *t*_acc_ = 150 s, scan rate = 0.100 V s^-1^, 0.05 M BR buffer solution, and pH 7

The intra-day precision of the stripping peak current signal (*I*_P_) was evaluated at three concentration levels of 4.0, 9.9, and 15.8 mg L^-1^, Figure 9. The experimentally obtained *I*_p_ and RSD values were compared with the corresponding 1/2RSD_H_ values calculated from the Horwitz equation [36].

The repeatability results showed that at *C*_SIB_ = 4.0 mg L^-1^, the relative standard deviation (RSD) was 3.95 % compared with 1/2RSD_H_ = 6.48 %. At *C*_SIB_ = 9.9 mg L^-1^, the RSD value was 3.46 %, lower than 1/2RSD_H_ = 5.63 %. Similarly, at *C*_SIB_ = 15.8 mg L^-1^, the obtained RSD value was 4.18 %, lower than 1/2RSD_H_ = 5.25 %. At all investigated SIB concentrations, the experimental RSD values were smaller than the corresponding 1/2RSD_H_ values, indicating that the proposed electrochemical method satisfies the repeatability requirement for *I*_p_ measurements.

#### Inter-day precision

**Figure 10.**
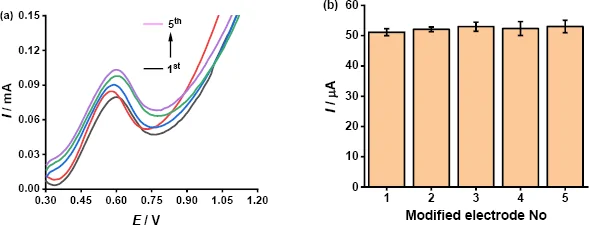
(a) Linear sweep voltammograms and (b) variation of the oxidation peak current (*I*_p_) of SIB obtained from five independently prepared AgNPs-ErGO/GCE modified electrodes

The inter-day precision of the proposed method was evaluated by independently preparing modified electrodes using the synthesized material and recording the voltammetric response of standard SIB solutions by the LSV technique on different days. After each measurement, the modified film on the electrode surface was completely removed by polishing the electrode with 0.03 μm Al₂O₃ powder, followed by re-modification according to the same fabrication procedure. This process was repeated five times under identical experimental conditions. The obtained results are presented in Figure 10.

#### Recovery

Standard SIB solutions were spiked into the placebo matrix at three concentration levels of 2.0, 3.9 and 5.9 mg L^-1^, followed by recording the corresponding voltammetric responses. The analysis was performed in triplicate at each concentration level. The accuracy of the proposed LSV method using the AgNPs-ErGO/GCE-modified electrode was evaluated based on recovery values (Rev, %) [41]. Subsequently, SIB was spiked into the samples at three concentrations of 1.976, 3.945 and 5.906 mg L^-1^. The quantitative determination of SIB was then carried out using the LSV method with the AgNPs-ErGO/GCE-modified electrode, and the results obtained are summarized in Table 3.

**Table 3.** Recovery results of the proposed LSV method using the AgNPs-ErGO/GCE modified electrode

Measurement No	Spiked *C*_SIB_ / mg L^-1^					
	1.976		3.945		5.906	
	Found *C*_SIB_ / mg L^-1^	Rev, %	Found *C*_SIB_ / mg L^-1^	Rev, %	Found *C*_SIB_ / mg L^-1^	Rev, %
1	1.779	90.03	3.502	88.78	6.107	102.4
2	1.828	92.52	3.782	95.88	6.089	103.1
3	1.817	91.94	3.724	94.39	5.461	92.48
Average	1.808	91.50	3.669	93.02	5.886	99.67
SD	0.026	1.303	0.148	3.741	0.368	6.227

The recovery values obtained for SIB at the three investigated concentration levels ranged from 88.78 to 103.1 %. These results meet the acceptable recovery criteria recommended by the AOAC guidelines (80 to 110 %) [41], indicating that the proposed method is accurate.

### Real sample analysis

The developed analytical method was applied to quantify sibutramine adulterated in several herbal-based weight-loss products collected from the market

A total of 30 herbal-based weight-loss products currently available in Vietnam were collected from different market sources. These samples included 24 capsules, 3 tablets, 1 powder, and 2 tea bags. The concentration of sibutramine in the samples was determined using the standard addition method. The SIB content, mg g^-1^ in each sample, was calculated according to Equation (9):



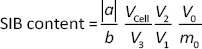

(9)


where *a* / μA and *b* / mg mL^-1^ are the intercept and slope of the linear regression equation, respectively; *V*_Cell_ = 10 mL is the volume of the electrochemical cell; *V*_0_ / mL is the volume of methanol used to dissolve the sample (solution A); *V*_1_ / mL is the aliquot volume of solution *A* taken for dilution; *V*_2_ / mL is the final volume after dilution (solution B); *V*_3_ / mL is the volume of solution B introduced into the electrochemical cell for analysis and *m*_0_ / g is the initial sample mass. The analytical results obtained from 30 collected herbal-based weight-loss products revealed that seven samples, namely #1, #2, #3, #4, #5, #6 and #7, were illegally adulterated with sibutramine. The proposed LSV method was applied for the determination of SIB in real samples, and the obtained results were compared with those from the reference HPLC-DAD method (Table 4).

**Table 4.** Determination of SIB content in seven positive samples using the proposed LSV method with the AgNPs-ErGO/GCE modified electrode and the HPLC-DAD method

Samples	Content, mg g^-1^	
	LSV	HPLC-DAD
#1	36.20	35.53
#2	42.77	42.80
#3	33.23	32.99
#4	66.61	64.83
#5	29.28	30.70
#6	24.41	23.72
#7	35.78	36.95

The results obtained from the proposed LSV method with the AgNPs-ErGO/GCE-modified electrode and the HPLC-DAD method were statistically compared using a paired-samples *t*-test, where *t* denotes the Student's t-statistic. The analysis revealed no statistically significant difference between the mean values obtained by the two methods (*t*(6) = 0.258, *p* = 0.805). These results demonstrate that the proposed LSV method based on the AgNPs-ErGO/GCE-modified electrode possesses satisfactory accuracy and reliability and can be considered a promising alternative analytical approach for determining SIB in herbal-based weight-loss products.

## Conclusion

A novel AgNPs-ErGO-based electrochemical sensor was successfully developed using a one-step electrochemical synthesis strategy, enabling the *in situ* formation and direct deposition of the nanocomposite onto the GCE surface. The resulting material exhibited a well-defined nanostructure with uniformly dispersed AgNPs and strong synergistic effects between AgNPs and ErGO, leading to enhanced electrochemical performance.

The modified electrode exhibited a large electroactive surface area, fast electron-transfer kinetics, and excellent electrocatalytic activity toward sibutramine oxidation. The electrochemical process was identified as an irreversible reaction involving equal proton and electron transfer, governed by a mixed diffusion-adsorption mechanism.

The developed LSV method exhibited satisfactory analytical performance, including low detection limit, good linearity, high precision, and acceptable recovery. Importantly, the method was successfully applied to real samples, revealing the presence of illegally adulterated sibutramine in herbal products. The results were consistent with those obtained by HPLC-DAD, demonstrating the reliability and practical applicability of the proposed sensor.

Overall, this work highlights the strong potential of the AgNPs-ErGO/GCE sensor as a rapid, sensitive and cost-effective alternative for quality control and safety monitoring of pharmaceutical and functional food products.

## Supplementary material

Additional data are available at https://pub.iapchem.org/ojs/index.php/admet/article/view/3433, or from the corresponding author on request.


