# Supplementary material for: Electrochemical detection of illicit sibutramine adulteration in herbal weight-loss products using an AgNPs-ErGO modified electrode
Source: ADMET DMPK. 2026 Jul 5;14:3433. doi: 10.5599/admet.3433 (PMC13499673; doi:10.5599/admet.3433)
Supplement: Supplementary file 2 [file ADMET-14-3433-S1.docx]

ADMET & DMPK **14** (2026) S3433

**Open Access : ISSN : 1848-7718**
[***http://www.pub.iapchem.org/ojs/index.php/admet***](http://www.pub.iapchem.org/ojs/index.php/admet)

Supplementary material to

Electrochemical detection of illicit sibutramine adulteration in herbal weight-loss products using an AgNPs-ErGO modified electrode

Nguyen Dang Thuy Anh^1,2^^<https://orcid.org/0009-0004-4844-0117>^, Vo Thi Cam Le^1^^<https://orcid.org/0009-0002-5743-8487>^, Huynh Van Chung^3^^<https://orcid.org/0000-0001-7519-1196>^,
Ngo Thi Thanh Xuan^4^^<https://orcid.org/0009-0000-9270-5056>^, Hoang Thi Minh Nguyet^5^^<https://orcid.org/0009-0008-2332-0214>^, Pham Thi Thanh Ha^2^^<https://orcid.org/0000-0003-2644-7620>^,
Ha Thuy Trang^6^^<https://orcid.org/0009-0004-5354-3954>^, Le Hoang Hao^1^^<https://orcid.org/0000-0002-7946-8698>^, Dao Thi Cam Minh^1^^<https://orcid.org/0000-0003-0786-3178>^, Nguyen Hai Phong^7^^<https://orcid.org/0000-0001-5354-1052>^
and Dinh Quang Khieu^7^^<https://orcid.org/0000-0003-3473-6377>^

^1^University of Medicine and Pharmacy, Hue University, Vietnam
^2^Hanoi University of Pharmacy, Vietnam
^3^Buon Ma Thuot Medical University, Vietnam
^4^Drug, cosmetic and food quality control center of Hue city, Vietnam
^5^Hue Central Hospital, Vietnam
^6^University of Education, Hue University, Vietnam
^7^University of Science, Hue University, Vietnam

ADMET & DMPK **14** (2026) 3433; <https://doi.org/10.5599/admet.3433>

**Figure S1.** The cyclic voltammograms and the corresponding linear relationships between anodic/cathodic peak currents and the square root of scan rate of three different electrodes including GCE (a-b),
GO/GCE (c-d), ErGO/GCE (e-f) in 5 mM [Fe(CN)₆]³⁻/⁴⁻ containing 0.1 M KCl used for estimating the electrochemically active surface area according to the Randles-Sevcik equation

**Figure S2.** The cyclic voltammograms and the corresponding linear relationships between anodic/cathodic peak currents and the square root of scan rate of three different electrodes including AgNPs/GCE (a-b), AgNPs-GO/GCE (c-d) and AgNPs-ErGO/GCE (e-f) in 5 mM [Fe(CN)₆]³⁻/⁴⁻ containing 0.1 M KCl used for estimating the electrochemically active surface area according to the Randles-Sevcik equation**.**

The electrochemically active surface area (ECSA) of the modified electrodes was estimated using the Randles-Ševčík equation for the reversible redox probe [Fe(CN)₆]³⁻/⁴⁻ at 5 mM in 0.1 M KCl. The ECSA values calculated from the anodic peak current for GCE, GO/GCE, ErGO/GCE, AgNPs/GCE, AgNPs-GO/GCE, and AgNPs-ErGO/GCE were 0.042, 0.007, 0.034, 0.037, 0.027, and 0.045 cm², respectively. Similar values were obtained from the cathodic peak current, namely 0.043, 0.007, 0.034, 0.039, 0.027 and 0.046 cm², respectively.

The accumulation potential (*E*_acc_) significantly affected the stripping signal of SIB (Figure S3). As *E*_acc_ increased from -0.3 to 0.0 V (*vs.* Ag/AgCl, 3 M KCl), the peak current increased and reached a maximum value of 44.32 ± 1.78 µA (RSD = 4.02 %, *n* = 3) at 0.0 V. Further increasing *E*_acc_ to +0.3 V caused a gradual decrease in the signal. Therefore, 0.0 V was selected as the optimal accumulation potential because it provided the highest and most well-defined stripping peak, favorable for improving analytical sensitivity and LOD determination.

**Figure S3.** (a) LSV curves of AgNPs-ErGO/GCE in 0.05 M BR (pH 7) containing 9.9 mg L^-1^ and (b) *I*_p_ values at different accumulation potentials (accumulation time: 120 s; scan rate: 0.100 V s^-1^; time step: 0.016 s; potential scan range from 0.3 to 1.2 V)

The accumulation time (*t*_acc_) significantly affected the stripping response of SIB (Figure S4). As *t*_acc_ increased from 30 to 180 s, the peak current increased linearly, while a further increase to 240 s caused the signal to approach saturation. Therefore, 150 s was selected as the optimal accumulation time because it provided a high analytical response while reducing the overall analysis time.

**Figure S4.** (a) LSV curves of AgNPs-ErGO/GCE in 0.05 M BR (pH 7) containing 9.9 mg L^-1^ and (b) *I*_p_ values at different accumulation times (accumulation potential: 0 V; scan rate: 0.100 V s^-1^; time step: 0.016 s;
potential scan range from 0.3 to 1.2 V)

As the scan rate increased from 0.010 to 0.300 V s⁻¹, the oxidation peak current of SIB increased remarkably, while the oxidation peak potential shifted toward more positive values, indicating an irreversible or quasi-reversible electrochemical process (Figure S5). At a scan rate of 0.100 V s⁻¹, the obtained peak current was sufficiently high for quantitative analysis, and the voltammetric peak still remained sharp and well-defined. Although higher scan rates produced larger signals, they also increased the capacitive current and broadened the peaks. Therefore, a scan rate of 0.100 V s⁻¹ was selected for subsequent experiments.

**Figure S5.** (a) LSV curves of AgNPs-ErGO/GCE in 0.05 M BR ( pH 7 ) containing 9.9 mg L^-1^ and (b) *I*_p_ values at different scan rates from 0.010 V to 0.300 V s^-1^ (accumulation potential: 0 V; accumulation time: 150 s;
time step: 0.016 s; potential scan range from +0.3 V to +1.2 V)
